# Comparison of a multiplex PCR with DNA barcoding for identification of container breeding mosquito species

**DOI:** 10.1186/s13071-024-06255-z

**Published:** 2024-04-02

**Authors:** Julia Reichl, Christina Prossegger, Sarah Petutschnig, Maria Sophia Unterköfler, Karin Bakran-Lebl, Mateusz Markowicz, Alexander Indra, Hans-Peter Fuehrer

**Affiliations:** 1 Institute for Medical Microbiology and Hygiene, Austrian Agency for Health and Food Safety (AGES), Vienna, Austria; 2 Institute of Parasitology, Department of Pathobiology, University of Veterinary Medicine Vienna, Vienna, Austria

**Keywords:** Multiplex PCR, *Aedes*, *Aedes albopictus*, Mosquitoes, Austria, Monitoring, Identification

## Abstract

**Background:**

Identification of mosquitoes greatly relies on morphological specification. Since some species cannot be distinguished reliably by morphological methods, it is important to incorporate molecular techniques into the diagnostic pipeline. DNA barcoding using Sanger sequencing is currently widely used for identification of mosquito species. However, this method does not allow detection of multiple species in one sample, which would be important when analysing mosquito eggs. Detection of container breeding *Aedes* is typically performed by collecting eggs using ovitraps. These traps consist of a black container filled with water and a wooden spatula inserted for oviposition support. *Aedes* mosquitoes of different species might lay single or multiple eggs on the spatula. In contrast to Sanger sequencing of specific polymerase chain reaction (PCR) products, multiplex PCR protocols targeting specific species of interest can be of advantage for detection of multiple species in the same sample.

**Methods:**

For this purpose, we adapted a previously published PCR protocol for simultaneous detection of four different *Aedes* species that are relevant for Austrian monitoring programmes, as they can be found in ovitraps: *Aedes albopictus*, *Aedes japonicus, Aedes koreicus,* and *Aedes geniculatus*. For evaluation of the multiplex PCR protocol, we analysed 2271 ovitrap mosquito samples from the years 2021 and 2022, which were collected within the scope of an Austrian nationwide monitoring programme. We compared the results of the multiplex PCR to the results of DNA barcoding.

**Results:**

Of 2271 samples, the multiplex PCR could identify 1990 samples, while species determination using DNA barcoding of the mitochondrial cytochrome c oxidase subunit I gene was possible in 1722 samples. The multiplex PCR showed a mixture of different species in 47 samples, which could not be detected with DNA barcoding.

**Conclusions:**

In conclusion, identification of *Aedes* species in ovitrap samples was more successful when using the multiplex PCR protocol as opposed to the DNA barcoding protocol. Additionally, the multiplex PCR allowed us to detect multiple species in the same sample, while those species might have been missed when using DNA barcoding with Sanger sequencing alone. Therefore, we propose that the multiplex PCR protocol is highly suitable and of great advantage when analysing mosquito eggs from ovitraps.

**Graphical Abstract:**

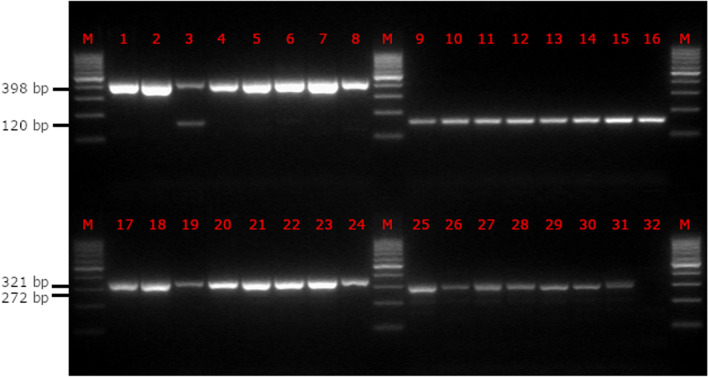

**Supplementary Information:**

The online version contains supplementary material available at 10.1186/s13071-024-06255-z.

## Background

Identification of mosquito species has become increasingly important in the last decades, due to the spread of (potentially) invasive species [1]. Container breeding mosquito species, which use natural or artificial containers for oviposition [2], are of particular concern due to their potential to be competent vectors for a variety of pathogens. The increase in global transport and traffic enables the introduction and spread of those container breeding mosquitoes worldwide while climate change and concomitant warmer temperatures facilitate establishment of stable populations [3, 4]. Additionally, container breeding *Aedes* mosquitoes can lay eggs that can withstand desiccation and cold temperatures, which allows overwintering in temperate regions, such as Europe [5–7]. In the last decades, three container breeding *Aedes* species have become particularly important for Europe, namely the Asian bush mosquito (*Aedes japonicus*), the Korean bush mosquito (*Aedes koreicus*) and the Asian tiger mosquito (*Aedes albopictus*).

*Aedes albopictus*, the Asian tiger mosquito, is the most relevant mosquito of these three for human and veterinary health. It is a competent vector for viruses, such as dengue, Zika and chikungunya [8–10], as well as for filarioid helminths, namely *Dirofilaria repens* and *Dirofilaria immitis* [11–14]. The native regions of *Ae.* *albopictus* are the tropical and subtropical Asian-Pacific regions from where it spread over all continents except the Antarctica [6, 8]. In Europe, the Asian tiger mosquito was first found in Albania in 1979 and later in Italy in 1990/1991. Since then, it has spread to more than 25 European countries [15–18].

*Aedes japonicus* originates from temperate regions of Eastern Asia [19] and was first reported in Europe in 2000 in northern France [20]. Under laboratory conditions, it is able to transmit viruses such as Japanese encephalitis virus [21, 22] and La Crosse virus [23], while West Nile virus has been identified multiple times in field-collected *Ae. japonicus* mosquitoes in the USA [24, 25]. Additionally, Austria reported the first natural infection of field-collected *Ae. japonicus* with Usutu virus in 2019 [26].

The Korean bush mosquito is native to Korea, Japan, north-eastern China and the far eastern parts of Russia [19, 27–29]. In 2008, *Ae. koreicus* was first found in Europe, in eastern Belgium [30]. Since then, a few other European countries, such as Italy [31, 32], Germany [33, 34], Switzerland [35], Hungary [36], the European part of Russia [37] and Austria [38] reported the presence of *Ae. koreicus*. The role of *Ae. koreicus* in transmission of pathogens remains unclear. However, studies suggest that this species is able to transmit Japanese encephalitis and *Dirofilaria immitis*, as well as some viruses such as chikungunya, under laboratory conditions [39–42].

In Austria, the first report of an exotic *Aedes* species showed the presence of *Ae.* *japonicus* in Styria in 2011 [43]. *Aedes japonicus* has been found in all provinces in Austria since its first report, has established stable populations, and can now be found in high numbers in all parts of Austria [44, 45]. *Aedes albopictus* was the second exotic *Aedes* species to be reported in Austria. It was first identified in Burgenland in 2012 [43]. After some initial single reports of *Ae. albopictus* mainly in Western Austria along the motorways, the number of reports has rapidly increased recently [38, 45]. In 2020, *Ae. albopictus* was first identified in Vienna, the capital city of Austria [46], and was found in the city of Graz in 2021 [47]. In 2022, the Asian tiger mosquito was found in every province in Austria if highway stations are included. However, stable populations are currently only documented in Vienna and Graz [48]. *Aedes koreicus* was first identified in Austria in 2017 in Carinthia [49]. Since then, there were some single reports in Carinthia, Styria and Tyrol [38, 48].

Identification of mosquitoes and their eggs greatly relies on morphological examination under a stereo microscope. Depending on the stage, some species might not be distinguishable with morphological methods. Therefore, it is important to incorporate molecular techniques for identification of mosquitoes [50–52]. DNA barcoding uses polymerase chain reaction (PCR) techniques to amplify conserved regions of the DNA with subsequent sequencing of the amplicon. The sequence obtained can then be compared with reference sequences in databases such as NCBI GenBank [53] to identify the mosquito species [54]. The most common DNA barcode is the mitochondrial cytochrome c oxidase subunit I (mtCOI) gene, which can serve as a molecular marker as it generally shows a low level of intra-species variation, but a high level of inter-species variation [54, 55]. While DNA barcoding using Sanger sequencing can additionally help with identifying cryptic species [56, 57], it does not allow accurate identification of multiple species in one sample [58]. Collection of *Aedes* eggs from container breeding species is typically performed by using so-called ovitraps, which consist of a black container filled with water and a wooden spatula plunged inside for oviposition support [44]. *Aedes* mosquitoes of different species might lay single or multiple eggs on the spatula. Morphological differentiation is possible but might be difficult depending on the species [59]. As molecular identification with Sanger sequencing of the mtCOI gene does not allow for identification of multiple species in one sample [58], multiplex PCR protocols targeting the specific species of interest can be of advantage for this purpose.

Bang et al. [60] designed a multiplex PCR protocol for simultaneous detection of six different *Aedes* species: *Ae. albopictus*, *Ae. koreicus*, *Ae. japonicus*, *Ae. flavopictus, Ae. togoi* and *Ae. hatorii*. As only the first three species are relevant for Austria, we adapted the PCR protocol and further developed it so that identification of an Austrian native *Aedes* species, *Ae. geniculatus*, was also possible. Ovitrap mosquito samples from the years 2021 and 2022 deriving from an Austrian nationwide monitoring program [48, 61] were used for evaluation of the adapted multiplex PCR protocol and a comparison with morphological identification and DNA barcoding of the mtCOI gene.

## Methods

### Mosquito sampling

*Aedes* eggs were collected over the course of 2 years (2021, 2022) within the scope of a nationwide mosquito monitoring program. Ovitraps, representing the ideal habitat for breeding of *Aedes* mosquitoes, were set up in all nine provinces of Austria from the beginning of May until the end of October in both years. Black plastic containers (1 L) filled with tap water (approximately 0.75 L) served as ovitraps. For oviposition possibility, a wooden spatula, fixed with a stainless-steel clamp, was inserted into the water. The wooden spatula and the water were exchanged on a weekly basis, and the removed spatula was sent to the laboratory for further analysis.

### Morphological analysis

Wooden spatulas were examined under the stereo microscope for the presence of mosquito eggs, more specifically *Aedes* eggs. Eggs were identified morphologically to species level, if possible, and subsequently removed from the spatula and collected in an Eppendorf tube (1.5 mL). Tubes were stored at −80 °C until molecular analysis was performed.

### DNA extraction

For molecular analysis, all eggs from each wooden spatula were homogenized using one ceramic bead (2.8 mm Precellys Ceramic Beads, VWR, Darmstadt, Germany) and a TissueLyser II (Qiagen, Hilden, Germany) as described previously [38]. DNA of the samples from 2021 was extracted by the University of Veterinary Medicine, Vienna using the innuPREP DNA Mini Kit (Analytik Jena, Jena, Germany) according to the manufacturer’s instructions. In 2022, molecular analyses were performed by the Austrian Agency for Health and Food Safety, which is why the protocol was changed and adapted to the available equipment at that laboratory. DNA isolation of the samples from 2022 was carried out with the BioExtract SuperBall Kit (Biosellal, Dardilly, France) on a KingFisher Flex96 robot (Thermo Fisher Scientific, Waltham, USA) following an in-house protocol.

### Multiplex PCR

The universal forward primer (Aedes-F), as well as the specific reverse primers for *Ae. albopictus* (ALB-R), *Ae. japonicus* (JAP-R) and *Ae. koreicus* (KOR-R), targeting the ribosomal ITS2 region were taken from the original protocol [60]. For *Ae. geniculatus*, we used all ITS2 reference sequences from the NCBI GenBank database to design a reverse primer (GEN-R) specific to that species and compatible with the other primers of the multiplex PCR. Primer design was performed with the software CLC Genomics Workbench 10 (Qiagen, Hilden, Germany). Primer sequences and details are shown in Table 1.

**Table 1:** Primers used for the multiplex PCR protocol

Primer	Sequence (5′–3′)	Forward/reverse	Source
Aedes-F	AGGACACATGAACACCCACA	Forward	[60]
ALB-R	GGAGCACACTGAGAGTTCCA	Reverse	[60]
JAP-R	TATACTACGCTGCCGAGAGG	Reverse	[60]
KOR-R	GCCTACTGATTGACGGGGTA	Reverse	[60]
GEN-R	ATGTATTCACCAACCGGG	Reverse	This study

PCR reactions consisted of 10 µL REDTaq Ready Mix (Merck, Darmstadt, Germany), 7 µL nuclease-free water, 0.4 µL of each primer (10 µM) and 1 µL of DNA, while the PCR conditions described by Bang et al. [60] were applied (94 ℃/5 min; then 94 ℃/30 s, 56 ℃/30 s, 72 ℃/30 s for 35 cycles; and the final extension at 72 ℃ for 5 min). Gel electrophoresis (gel: 2.0% agarose gel in 0.5× TBE buffer; voltage: 100 V; run time: 90–120 min) and visualisation with the Gel Doc 2000 (Bio-Rad Laboratories, Hercules, USA) was performed to identify potential PCR products. The GeneRuler 100 bp DNA Ladder (Thermo Fisher Scientific, Waltham, USA) was used for sizing of PCR products. For confirming correct PCR conditions and successful primer binding, the PCR was first evaluated with representative DNA samples of the four relevant *Aedes* species (identified by DNA barcoding and morphological analysis) before the protocol was applied to the collected samples. The representative samples included *Ae. koreicus* larvae from the Viennese Central Cemetery [62], while the egg samples of the other species were obtained during the nationwide monitoring program in Austria [48, 61].

### DNA barcoding

DNA barcoding of the mitochondrial cytochrome oxidase subunit I (mtCOI) gene was performed as previously described [56]. In 2021, the PCR products amplified with the primers LepF1 (5′-ATTCAACCAATCATAAAGATAT-3′) and LepR1 (5′-TAAACTTCTGGATGTCCAAAAA-3′) were sequenced at LGC Genomics GmbH, Berlin, Germany.

In 2022, PCR amplification was performed at the Austrian Agency for Health and Food Safety with the primers M13F-LepF1 (5′-TGTAAAACGACGGCCAGATTCAACCAATCATAAAGATAT-3′) and M13R-LepR1 (5′-CAGGAAACAGCTATGACTAAACTTCTGGATGTCCAAAAA-3′). M13 sequences were added to the primers (without resulting in different primer binding properties) for facilitating the subsequent workflow. The PCR conditions of the protocol using the primers LepF1 and LepR1 were applied [56]. For PCR cleanup, 2 µL of the ExoSAP-IT Express reagent (Applied Biosystems, Waltham, USA) were mixed with 5 µL PCR product and incubated at 37 °C for 4 min and 80 °C for 1 min. Subsequent cycle sequencing reactions with the sequencing primer M13R (5′-CAGGAAACAGCTATGAC-3′) were set up using the BigDye Terminator v3.1 Cycle Sequencing kit (Applied Biosystems, Waltham, USA). The reactions contained 8 µL BigDye Terminator 3.1 Ready Reaction Mix, 9.68 µL ddH2O, 0.32 µL of the sequencing primer M13R (10 µM) and 2 µL of template. The conditions for the cycle sequencing were chosen as recommended by the manufacturer. After cycle sequencing, the reactions were purified by adding 90 µL of SAM solution (Applied Biosystems, Waltham, USA) and 20 µL of the XTerminator solution (Applied Biosystems, Waltham, USA). After shaking for 20 min at 1800 rpm on an Eppendorf MixMate (Eppendorf, Hamburg, Germany) and centrifugation at 1000*g* for 2 min, the sequencing reactions were analysed with an ABI Genetic Analyzer 3500 (PA Protocol: BDTv3.1_PA_Protocol_POP7, Basecall Version: KB 1.4.1.8) (Applied Biosystems, Waltham, USA). After analysis, the resulting sequences were compared to sequences of two public databases (NCBI Genbank, www.ncbi.nlm.nih.gov/genbank; BOLD Systems, www.boldsystems.org) for species identification.

### Data analysis

All data obtained was documented in Microsoft Excel (Microsoft, Washington, USA) and analysed using the software R version 4.2.3 [63].

## Results

### Confirmation of PCR conditions and primer binding

**Fig. 1:**
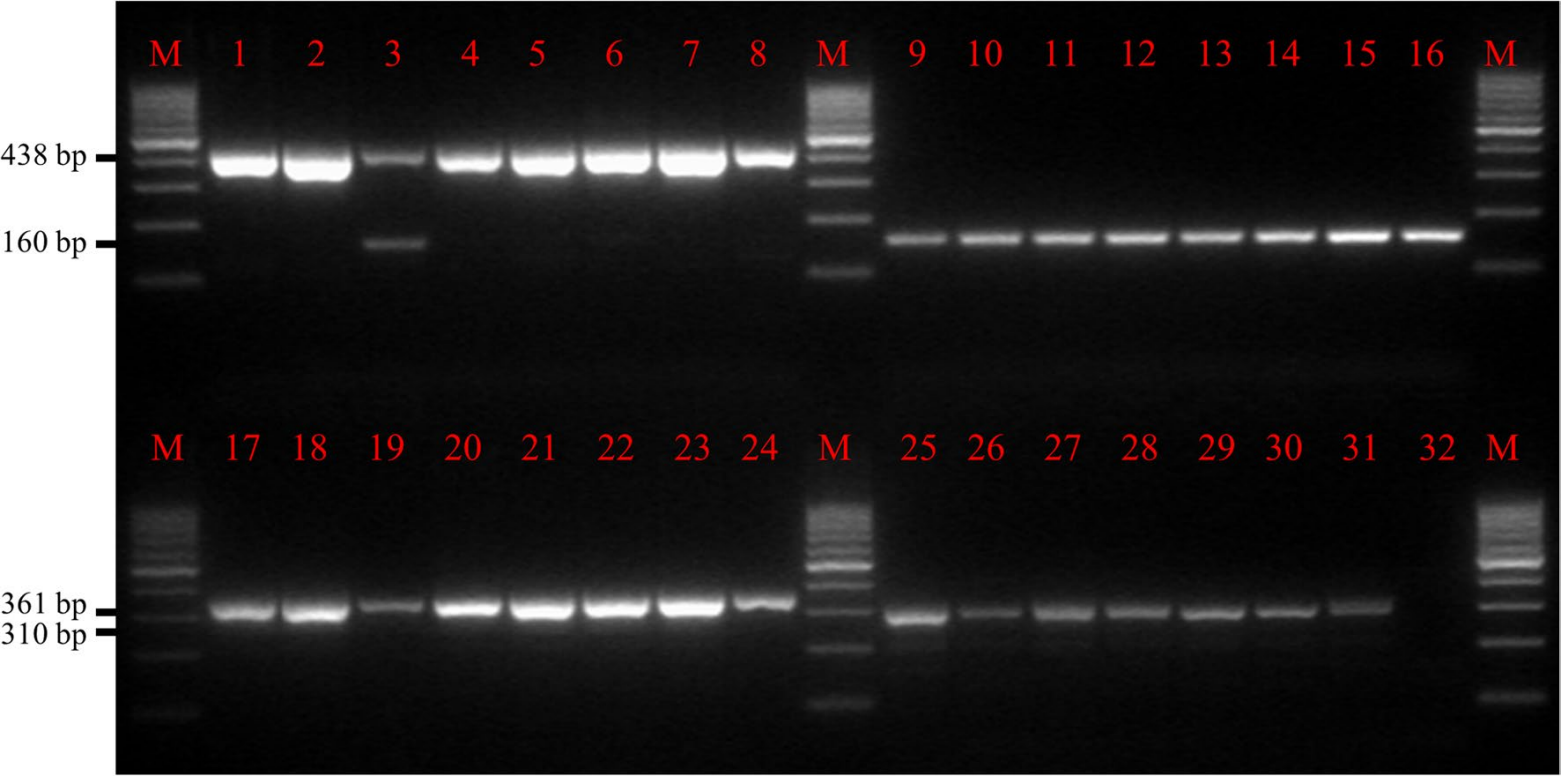
Results of the multiplex PCR of representative DNA samples of the four relevant *Aedes* species. The 31 DNA samples (lanes 1–8: *Ae. albopictus* eggs; lanes 9–16: *Ae. japonicus* eggs; Lanes 17–24: *Ae. koreicus* larvae; lanes 25–31: *Ae. geniculatus* eggs) were previously identified morphologically and with DNA barcoding and subsequently analysed using the multiplex PCR protocol. All samples show specific PCR products of the correct sizes according to the expected species: 438 bp for *Ae. albopictus*, 361 bp for *Ae. koreicus*, 310 bp for *Ae. geniculatus* and 160 bp for *Ae. japonicus*. The sample in lane 3 (expected to be *Ae. albopictus*) shows an additional PCR product at 160 bp, indicating the presence of *Ae. japonicus*

For a first evaluation, a total of 31 DNA samples from ovitraps and larvae (8 *Ae. koreicus* larval samples, eight *Ae. albopictus* egg samples, eight *Ae. japonicus* egg samples, seven *Ae. geniculatus* egg samples) and one negative control (water) were analysed using the adapted multiplex PCR protocol. The samples were previously identified morphologically and by DNA barcoding. After gel electrophoresis (Fig. 1), all samples showed specific PCR products of the correct sizes according to the expected species (438 bp for *Ae. albopictus*, 361 bp for *Ae.* *koreicus*, 310 bp for *Ae. geniculatus* and 160 bp for *Ae. japonicus*). One sample, which was expected to be *Ae. albopictus*, showed an additional PCR product at 160 bp, indicating the presence of *Ae. japonicus*.

### Analysis of ovitrap samples from 2021 and 2022

In total, 2271 samples from the years 2021 and 2022 were analysed using morphology, the adapted multiplex PCR and DNA barcoding with Sanger sequencing. Morphological specification of the eggs revealed *Ae. albopictus* in 203 samples and *Ae. geniculatus* in 122 samples, while 104 samples were either classified as another species or not identified at all. Differentiation of *Ae. japonicus* and *Ae. koreicus* was not possible when morphologically looking at egg samples, which is why 1750 samples were classified as *Ae. koreicus*/*Ae. japonicus*. In an additional 92 samples, morphological examination revealed the presence of more than one species (5 *Ae. albopictus*/*Ae. geniculatus*, 8 *Ae. albopictus*/*Ae. japonicus*/*Ae. koreicus*, 79 *Ae. geniculatus*/*Ae. japonicus*/*Ae. koreicus*).

The results of the multiplex PCR analyses identified 162 *Ae. albopictus*, 1705 *Ae. japonicus* and 76 *Ae. geniculatus* samples. In those samples, DNA of only one species was present. 281 samples could not be identified, as no PCR product was visible on the gel. *Aedes koreicus* was not detected in any of the analysed samples. According to the multiplex PCR, DNA of multiple species was present in 47 samples. Of those, 18 were mixtures of *Ae. albopictus* and *Ae. japonicus*, while 29 samples contained DNA of *Ae. japonicus* and *Ae. geniculatus*.

DNA barcoding of mtCOI detected 163 *Ae. albopictus*, 88 *Ae. geniculatus* and 1281 *Ae. japonicus*, while *Ae. koreicus* was not identified. A total of 190 samples were classified as another species or organism. In 549 samples, DNA barcoding did not show any results due to low sequencing quality or lack of amplification of mtCOI. With DNA barcoding using Sanger sequencing, mixtures of different species could not be detected (Table 2).

**Table 2:** Contingency tables for the three species *Ae. albopictus*, *Ae. japonicus* and *Ae. geniculatus*

		Multiplex PCR		
DNA barcoding	*Aedes albopictus*	Positive	Negative	Total
	Positive	144	19	163
	Negative	36	2072	2108
	Total	180	2091	2271
	*Aedes japonicus*	Positive	Negative	Total
	Positive	1251	30	1281
	Negative	501	489	990
	Total	1752	519	2271
	*Aedes geniculatus*	Positive	Negative	Total
	Positive	68	20	88
	Negative	37	2146	2183
	Total	105	2166	2271

The table shows the amounts of samples positive or negative for the individual species, analysed with multiplex PCR and DNA barcoding

To compare the identification of container-breeding mosquitoes with DNA barcoding to the multiplex PCR protocol, contingency tables for the species *Ae. albopictus*, *Ae. japonicus* and *Ae. geniculatus* were created (Table 2). Due to the absence of *Ae. koreicus* in the analysed ovitrap samples, it was not possible to compare the two methods for this species. Percent agreement and Cohen’s Kappa coefficients (*κ*) were calculated to demonstrate the agreement between the two applied methods for each parameter/species: *Ae. albopictus* 97.58%, *κ* = 0.83 [95% confidence interval (CI) 0.78–0.87]; *Ae. japonicus* 76.62%, *κ* = 0.50 (95% CI 0.46–0.53); *Ae. geniculatus* 97.49%, *κ* = 0.69 (95% CI 0.62–0.77).

Samples that, according to the multiplex PCR, contained DNA of *Ae. albopictus* and *Ae. japonicus* (*n* = 18), were identified as *Ae. albopictus* in 10 cases and as *Ae. japonicus* in two cases when using DNA barcoding. Six of those samples showed no result with Sanger sequencing. While the multiplex PCR detected *Ae. japonicus* and *Ae. geniculatus* (*n* = 29), DNA barcoding showed no result (*n* = 4), *Ae. japonicus* (*n* = 8), *Ae. geniculatus* (*n* = 15) or another organism (*n* = 2).

The comparison of the molecular techniques with morphology was out of the scope of this study. However, to be able to better evaluate the performance of the two methods, additional contingency tables including morphological results were created for each species (Additional file 1: Tables S1, S2).

## Discussion

DNA barcoding using Sanger sequencing offers the possibility to identify a wide variety of different mosquito species (and other organisms). However, in contrast to a multiplex PCR, the method is not suitable for detection of multiple species in one sample. In a total of 47 samples, Sanger sequencing either detected only one species or did not show any result, while the multiplex PCR revealed the presence of multiple species. In 8 of 18 cases, *Ae. albopictus* was not identified in a sample containing more than one species when using DNA barcoding. Based on the results of this study, it can be assumed that using DNA barcoding with Sanger sequencing can lead to an under-reporting when dealing with samples that might contain DNA of several species. Hence, we propose that the described multiplex PCR protocol is of great advantage for certain monitoring purposes (e.g. ovitrap monitoring).

A Cohen’s kappa coefficient between 0.40 and 0.59 indicates weak agreement, while values from 0.60–0.79 to 0.80–0.90 are to be interpreted as moderate and strong agreement, respectively [64]. Based on the Cohen’s kappa coefficients, the agreement between the two methods for detecting *Ae. albopictus* is quite high, which is also shown by the percent agreement (97.58%). In contrast, Cohen’s kappa coefficient of *Ae. japonicus* (*κ* = 0.50) indicates only little agreement between the PCR and DNA barcoding, demonstrated also by a lower percent agreement of 76.62%. The results further indicate moderate agreement between the two methods when looking at the identification of *Ae. geniculatus*, with a Cohen’s kappa coefficient of 0.69 and a percent agreement of 97.49%. Differences in the degree of agreement between the species might be caused due to possible biases (primer bias, PCR selection, PCR drift), which result in higher amplification of some templates due to their properties (e.g. GC content and gene copy numbers) [65]. Another reason might be the difference in sample size, as a considerably higher number of samples positive for *Ae. japonicus* was analysed. Additionally, *Ae. japonicus* is considered a species complex, which is (currently) composed of four subspecies [66]. PCR and/or sequencing primers could show different binding properties related to the different subspecies, affecting the overall performance of those methods. Recently, it has been suggested that *Ae. geniculatus* either has cryptic sibling species or that it is also a species complex comprised of multiple subspecies [67]. However, this still needs to be further investigated since currently there are only few DNA sequences available in public databases for this species. This also posed a challenge for designing the primer for *Ae. geniculatus* in this study.

When comparing the number of negative results (no species identification), the multiplex PCR performed better than DNA barcoding. A total of 281 samples could not be identified by PCR, while DNA barcoding with Sanger sequencing did not lead to a result in 549 cases. Inspection of the contingency table (Table 2) makes it clear that the multiplex PCR generally performed slightly better in identifying the relevant container-breeding *Aedes* species. The biggest difference in performance can be observed with *Ae. japonicus*. A total of 501 PCR positive samples could not be identified as *Ae. japonicus* by Sanger sequencing, while only 30 *Ae. japonicus* samples would have been overlooked when using the multiplex PCR. Other organisms (e.g. Ceratopogonidae eggs or Amoebozoa such as *Vanella simplex*), which can also be detected during DNA barcoding, might be present in some of the samples and thereby lower the detection rate of sequencing when looking at the four relevant *Aedes* species.

While the aim of this study was primarily to compare the two molecular methods with each other, morphological results were included for a better overview and evaluation of the techniques. Generally, morphological analysis resulted in less non-identifiable samples compared to the multiplex PCR and DNA barcoding. A reason for the weaker performance of the molecular techniques might be that there is not enough DNA present (e.g. in samples with few eggs or after errors during DNA extraction). Nonetheless, some samples would have been overlooked when using morphological inspection alone (especially if scientists are not experienced in morphological specification of mosquito eggs). Therefore, we suggest complementing morphological assessment with molecular techniques to decrease mis- or non-identifications. Due to its better performance and higher agreement with morphology, we propose to use the multiplex PCR as a second method for identification of eggs from ovitrap samples. The analysed ovitrap samples from 2021 to 2022 did not include any *Ae. koreicus* eggs. While larvae of *Ae. koreicus* were found in the Viennese Central Cemetery in 2021, ovitraps in the same region were negative for this species. Possible reasons for the absence of eggs might be that there were too few *Ae. koreicus* females present in the area or that they preferred other breeding sites [62]. A comparison between Sanger sequencing and the multiplex PCR protocol was therefore not possible for this species and needs to be investigated in further experiments. However, the proposed PCR protocol was validated with representative *Ae. koreicus* larval samples. As species-specific bands were observed on the agarose gel, it is highly likely that the PCR protocol is also suitable for identification of *Ae. koreicus* in ovitrap samples.

Differentiation of *Ae. koreicus* and *Ae. geniculatus* might be difficult in some cases, as the PCR products lie close together when being visualised by gel electrophoresis. It is therefore important to ensure that the run time of the gel electrophoresis is adequate (in our cases 1.5–2 h). Additionally, results of other methods (e.g. morphology) can be taken into account for evaluation of the PCR results. If interpretation of the results concerning *Ae. koreicus* and *Ae. geniculatus* is still not possible, a second PCR using the universal forward primer and only one reverse primer (specific to *Ae. koreicus* or *Ae. geniculatus*) can be carried out for confirmation. Alternatively, agarose gel electrophoresis can be substituted by a fragment analysis using a suitable instrument (e.g. ABI 3500 Genetic Analyzer, Applied Biosystems, Waltham, USA) and fluorescently labelled primers. Fragment analysis allows for separation of PCR products that show a size difference of only 1–2 nt and can thereby improve analysis and interpretation of multiplex PCR reactions. Furthermore, the protocol can be adapted for analysis with a real-time PCR (RT–PCR) machine using fluorescently labelled primers or probes. Additionally, a RT–PCR approach offers higher sensitivity for detecting the four species and provides results more quickly than a conventional PCR as there is no need for subsequent gel electrophoresis. Additionally, PCR products can be quantified when using RT–PCR [68]. In the future, mosquito species identification will most probably benefit from the recent emergence of next-generation sequencing applications. Using NGS technology, DNA metabarcoding can be performed, which allows detection of multiple species in the same sample while being able to identify a wide range of different species. At the moment, however, PCR approaches still score with less time-consuming protocols and fewer costs, when compared with NGS applications. Nonetheless, further development of new methods for mosquito identification (and pathogen detection) would likely improve current workflows.

## Conclusions

The multiplex PCR protocol described in this study can serve as a powerful tool for large-scale analysis of container-breeding mosquitoes. Especially when detection of multiple species in the same sample might be necessary (e.g. ovitrap samples), the multiplex PCR is more suitable than DNA barcoding with Sanger sequencing. Additionally, PCR analyses are typically less time-consuming and cheaper compared to sequencing technologies. Therefore, we propose that this multiplex PCR protocol is highly suitable and of great advantage for analysing container-breeding mosquitoes.

### Supplementary Information

**Additional file 1: Table S1.** Contingency table for the three species *Ae. albopictus*, *Ae. japonicus* and *Ae. geniculatus* for comparison of the multiplex PCR and morphological analysis. The samples positive or negative for the individual species, when examined morphologically and by the multiplex PCR, are depicted in this table. **Table S2.** Contingency table for the three species *Ae. albopictus*, *Ae. japonicus* and *Ae. geniculatus* for comparison of DNA barcoding and morphological analysis. The amount of positive and negative samples after DNA barcoding and morphological examination are shown in this table.

## Data Availability

The datasets used and/or analysed during the current study are included in this published article (and its supplementary information files).
