# Supplementary material for: Comparison of a multiplex PCR with DNA barcoding for identification of container breeding mosquito species
Source: Parasit Vectors. 2024 Apr 2;17:171. doi: 10.1186/s13071-024-06255-z (PMC10985852; doi:10.1186/s13071-024-06255-z)
Supplement: Supplementary file 1 — Additional file 1: Table S1. Contingency table for the three species Ae. albopictus, Ae. japonicus and Ae. geniculatus for comparison of the multiplex PCR and morphological analysis. The samples positive or negative for the individual species, when examined morphologically and by the multiplex PCR, are depicted in this table. Table S2. Contingency table for the three species Ae. albopictus, Ae. japonicus and Ae. geniculatus for comparison of DNA barcoding and morphological analysis. The amount of positive and negative samples after DNA barcoding and morphological examination are shown in this table. [file 13071_2024_6255_MOESM1_ESM.docx]

**Supplementary** **Material**

**Table S1 Contingency table for the three species *Ae. albopictus*, *Ae. japonicus* and *Ae. geniculatus* for comparison of the multiplex PCR and morphological analysis.** The samples positive or negative for the individual species, when examined morphologically and by the multiplex PCR, are depicted in this table.

|  |  | **Multiplex PCR** | | |
| --- | --- | --- | --- | --- |
| **Morphological analysis** | *Aedes albopictus* | Positive | Negative | Total |
|  | Positive | 153 | 63 | 216 |
|  | Negative | 27 | 2028 | 2055 |
|  | Total | 180 | 2091 | 2271 |
|  |  |  |  |  |
|  | *Aedes japonicus* | Positive | Negative | Total |
|  | Positive | 1648 | 189 | 1837 |
|  | Negative | 104 | 330 | 434 |
|  | Total | 1752 | 519 | 2271 |
|  |  |  |  |  |
|  | *Aedes geniculatus* | Positive | Negative | Total |
|  | Positive | 96 | 110 | 206 |
|  | Negative | 9 | 2056 | 2065 |
|  | Total | 105 | 2166 | 2271 |

**Table S2 Contingency table for the three species *Ae. albopictus*, *Ae. japonicus* and *Ae. geniculatus* for comparison of DNA barcoding and morphological analysis.** The amount of positive and negative samples after DNA barcoding and morphological examination are shown in this table.

|  |  | **DNA barcoding** | | |
| --- | --- | --- | --- | --- |
| **Morphological analysis** | *Aedes albopictus* | Positive | Negative | Total |
|  | Positive | 151 | 65 | 216 |
|  | Negative | 12 | 2043 | 2055 |
|  | Total | 163 | 2108 | 2271 |
|  |  |  |  |  |
|  | *Aedes japonicus* | Positive | Negative | Total |
|  | Positive | 1207 | 630 | 1837 |
|  | Negative | 74 | 360 | 434 |
|  | Total | 1281 | 990 | 2271 |
|  |  |  |  |  |
|  | *Aedes geniculatus* | Positive | Negative | Total |
|  | Positive | 85 | 121 | 206 |
|  | Negative | 3 | 2062 | 2065 |
|  | Total | 88 | 2183 | 2271 |
